# Inflammation as a Risk of Developing Chronic Kidney Disease in Rheumatoid Arthritis

**DOI:** 10.1371/journal.pone.0160225

**Published:** 2016-08-18

**Authors:** Masako Kochi, Kentaro Kohagura, Yoshiki Shiohira, Kunitoshi Iseki, Yusuke Ohya

**Affiliations:** 1 Department of Cardiovascular Medicine, Nephrology and Neurology, University of the Ryukyus School of Medicine, Nishihara, Okinawa, Japan; 2 Yuuaikai Nanbu Hospital, Itoman, Okinawa, Japan; 3 Dialysis Unit, University Hospital of the Ryukyus, Nishihara, Okinawa, Japan; 4 Yuuaikai Tomishiro Central Hospital, Tomigusuku, Okinawa, Japan; 5 Okinawa Heart and Renal Association, Naha, Okinawa, Japan; Hospital Universitario de la Princesa, SPAIN

## Abstract

**Objective:**

The relationship between chronic inflammation and the incidence of chronic kidney disease (CKD) remained not-clear in patients with rheumatoid arthritis (RA). This study aims to examine the relationship between persistently high C-reactive protein (CRP), a marker of inflammation, and the incidence of CKD in RA.

**Methods:**

We retrospectively examined the relationship between the levels of CRP and incidence of CKD in 345 RA patients. The outcome of interest was incidence of CKD, defined as an estimated glomerular filtration rate (eGFR) <60 mL/min/1.73 m^2^ and/or positive dipstick testing for proteinuria for ≥3 months. We defined high CRP, as >3.0 mg/L. On the basis of three measurements of CRP for 6-months period, patients were divided into three groups: group 1, including patients with no high CRP values; group 2, patients with transient high CRP values (once or twice) and group 3, patients with persistently high CRP values.

**Results:**

During a median follow-up period of 89 months, 14% of all patients developed CKD. The cumulative incidence of CKD was 7% in group 1, 14% in group 2 and 22% in group 3 (P = 0.008, log-rank test). In a multivariate analysis, including classical risk factors for CKD, persistently high CRP was an independent predictor of the incidence of CKD (hazard ratio, 3.00; 95% confidence interval, 1.23–8.53; P = 0.01).

**Conclusions:**

Persistently high CRP was a significant risk factor for the incidence of CKD. Results suggest that persistent inflammation is a marker for the high risk of CKD in RA.

## Introduction

Chronic kidney disease (CKD) is common[1–5] in patients with rheumatoid arthritis (RA) which is a chronic, immune-mediated, systemic inflammatory disease. Several conditions such as secondary amyloidosis, glomerulonephritis or a drug-related cause could explain the relationship between CKD and RA[6, 7]. However, specific causes of CKD generally are not determined among most patients with RA. Thus, it is critically important to elucidate the risk factor for the incidence of CKD among them.

C-reactive protein (CRP), a marker of inflammation, is associated with RA disease activity; a persistently active form of the disease is characterised by elevated CRP levels and is associated with a more rapid radiological progression[8, 9]. It has been suggested that achieving clinical remission or low disease activity, defined as abrogation of inflammation within 6 months after starting treatment, is important for maintaining better articular function[10, 11]. In addition, an association between the degree of inflammation and an increased risk for cardiovascular, pulmonary, liver and skeletal diseases has been reported in patients with RA[12]. However, it remains to be determined whether inflammation is a risk factor for the incidence of CKD. It seems to be clinically important to elucidate whether the degree of inflammation in short-term period may have an impact on renal outcome; however, earlier resolution of inflammation has been recommended for better articular outcome.

Therefore, in the present study, we examined the relationship between persistent elevation of CRP during 6 months and the incidence of CKD in patients with RA.

## Methods

### Participants

**Fig 1:**
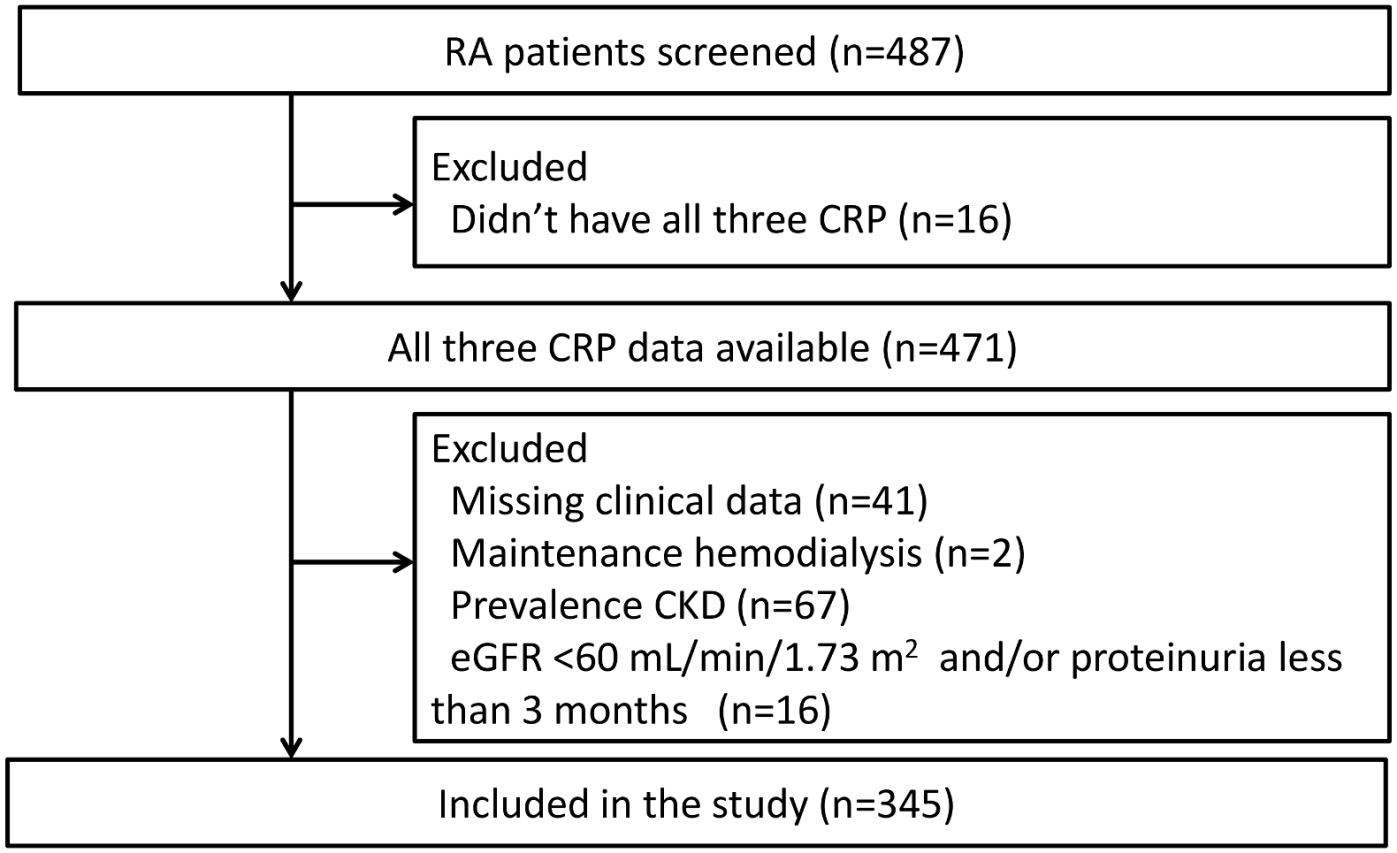
Flow chart of the study population. This chart indicates the selection of the study population and the exclusion strategy.

We conducted a retrospective study using digital medical records from Tomishiro Central Hospital (Okinawa, Japan) after obtaining a permissions of Ethical Committee of Tomishiro Central Hospital. The data from medical records were anonymized before access for this study. As a baseline for this study, we screened 487 adult patients (≥18 years of age) who visited the hospital in April 2006 and fulfilled the 1987 American College of Rheumatology criteria for RA[13]. Of these patients, we excluded those who did not have all three CRP measurement (n = 16), those with missing clinical data (n = 41), those receiving maintenance hemodialysis (n = 2), those with CKD (n = 67), and those with existing eGFR <60 mL/min/1.73 m^2^ and/or proteinuria for less than three months at baseline for this study (n = 16; Fig 1). Finally, we included 345 patients and reviewed retrospectively the incidence of CKD, death and lost to follow-up until the end of the study (31 March 2014) and examined the relationship between persistent elevation of CRP and risk of CKD.

### Data collection

At outpatient department, nurses or doctors dispensed a lifestyle and medical history questionnaire, collected blood and urine samples and measured blood pressure levels. Classical CKD risk factors at baseline for this study were hypertension (defined as more than two resting blood pressure measurements of ≥140 mm Hg systolic and/or ≥90 mm Hg diastolic or treatment with antihypertensive agents), diabetes mellitus (defined as at least two measurements of fasting plasma glucose level of ≥126 mg/dL, a 2-h plasma glucose level of ≥200 mg/dL or treatment with hypoglycemic agents) and dyslipidemia (defined as a low-density lipoprotein cholesterol level of ≥140 mg/dL, high-density lipoprotein cholesterol level of <40 mg/dL, triglyceride level of ≥150 mg/dL or treatment with specific lipid-lowering agents, according to the criteria of the Japan Atherosclerosis Society[14]). Baseline height and weight were recorded and body mass index (BMI) was calculated. Overweight was defined as a BMI of ≥25 kg/m^2^. We also analysed clinical characteristics of RA, including RA duration, and the continuous use of anti-rheumatic medication for at least 6 months during the period of the first to third measurement of CRP for this study [methotrexate (MTX); other disease modifying anti-rheumatic drugs (DMARDs), including bucillamine, sulfasalazine, D-penicillamine, auranofin, actarit, mizoribine, tacrolimus and leflunomide; biological agents; corticosteroids and nonsteroidal anti-inflammatory drugs (NSAIDs)] based on medical records. In DMARDs, nephrotoxic DMARDs were defined as drugs which have potential renal toxicity include the following: bucillamine, D-penicillamine, auranofin and tacrolimus. Serum creatinine level was measured using the enzymatic method (Sekisui Medical, Tokyo, Japan).

Estimated glomerular filtration rate (eGFR) was calculated using the Japanese Society of Nephrology formula as follows[15]:

$$\text{eGFR }(\text{mL}/\text{min}/1.73{\text{ m}}^{2})\;=\;194\;\times {\text{ serum creatinine}}^{-1.094}\times {\text{ age}}^{-0.287}\times \;0.739\;\left(\text{if female}\right).$$

Proteinuria was defined as a dipstick urinalysis score of 1+ or more (Eiken Chemical, Tokyo, Japan). CRP levels were measured using the latex agglutination immunoassay method using the Nanopia CRP (Sekisui Medical, Tokyo, Japan). The normal range of this assay was ≤3 mg/L. Measurements of blood glucose, total cholesterol, low-density lipoprotein cholesterol, high-density lipoprotein cholesterol and triglycerides were obtained using a HITACHI 7170 autoanalyser (Hitachi, Tokyo, Japan). Rheumatoid factor was measured using the latex agglutination test (Mitsubishi Kagaku Iatron, Tokyo, Japan).

### Subgroups by CRP data

In this study, we used CRP as a marker of inflammation. High CRP was defined as >3.0 mg/L. To diagnose persistently high CRP, we chose CRP at three time points: at baseline for this study, at 3 months (range of 2–4 months) and at 6 months (range of 5–7 months) from the baseline for this study. On the basis of CRP measurements, patients were divided into three groups: group 1, those with no high CRP values (defined as all three CRP values ≤3.0 mg/L); group 2, those with transient high CRP values (defined as once or twice CRP values >3.0 mg/L) and group 3, those with persistently high CRP values (defined as all three CRP values >3.0 mg/L).

### Outcome

CKD was defined as an eGFR <60 mL/min/1.73 m^2^ and/or proteinuria for ≥3 months, according to the criteria of the Japanese Society of Nephrology. Incidence of CKD was defined as the new development of CKD.

### Statistical analyses

Parametric analyses were performed by analysis of variance with the Tukey–Kramer test as a post hoc test to analyze differences in discrete variables between groups. Nonparametric analyses were performed by Kruskal–Wallis test with the Steel–Dwass test. Categorical variables were examined with the Pearson’s chi–square test. Cumulative incidence rates were calculated using the Kaplan–Meier method, and differences between groups were assessed using the log-rank test. Patients were censored at time of event, at death, at lost to follow-up, or at the end of follow-up, whichever came first. The Cox proportional hazard analysis was used to calculate hazard ratios (HRs) and 95% confidence intervals (CIs) of developing CKD in crude and adjusted models. We used four models of multivariate analysis to examine the association between the incident CKD and persistent elevation of CRP. Covariates included in the adjusted models were as follows: Model 1) age (every 10 years), sex, and CRP subgroups; Model 2) covariates included in model 1 plus baseline eGFR (+5mL/min/1.73 m^2^); Model 3) covariates included in mode2 plus classical risk factors for CKD (hypertension [yes/no], diabetes mellitus [yes/no], dyslipidemia [yes/no], overweight [yes/no], and smoking [yes/no]); Model 4) covariates included in model 3 plus baseline medication use which may have a toxic effect on the kidney (MTX[yes/no], nephrotoxic DMARDs [yes/no], and NSAIDs [yes/no]). The time starts after the third measurement of CRP for the cumulative incidence rates and Cox proportional hazard analysis. The relationship between the median CRP values along the entire follow-up and the median CRP values during the first 6 months of follow-up was examined using Pearson’s correlation coefficient with log-transformation of variables because of non-normal distribution of values. Receiver operating characteristic (ROC) analysis was conducted to determine the cut-off value of the median CRP values along the entire follow-up that predicts incidence of CKD. Analyses were performed using the JMP software package (SAS Institute Inc., Cary, NC, USA). Continuous data are expressed as mean ± standard deviation, and skewed distributions are presented as the median with 25^th^ and 75^th^ percentiles. P < 0.05 was considered statistically significant.

## Results

### Baseline characteristics

Baseline characteristics of the patients are summarised in Table 1. The mean age was 57.2 years and 294 (85%) of the patients were female. The mean eGFR was 86.9 mL/min/1.73 m^2^. For the study, patients were classified into three groups by CRP changes: 101 (29%) were included in group 1 (remained normal CRP in 6 months), 100 (29%) in group 2 (transient high CRP values) and 144 (42%) in group 3 (persistently high CRP values). The CRP values were significantly higher in groups 2 and 3 than those in group 1 at all three points. The prevalence of rheumatoid factor-positive, methotrexate, corticosteroids and NSAIDs use were higher in groups 2 and 3 than group 1. Patients in group 3 had significantly higher CRP values at all three time points and had a higher prevalence of hypertension than groups 1 and 2. There were no significant differences in eGFR among the three groups at the baseline.

**Table 1:** Baseline clinical characteristics of patients.

		CRP category			
Characteristic	Total (n = 345)	Group1^a^ (n = 101)	Group2^b^ (n = 100)	Group3^c^ (n = 144)	*P* value
**Age, years**	57.2 (12.8)	53.0 (12.1)	57.9 (13.7)	58.9^d^(12.4)	0.009
**Female, n (%)**	294 (85)	90 (89)	84 (84)	120 (83)	0.42
**Serum creatinine, mg/dL**	0.59 (0.10)	0.61 (0.09)	0.60 (0.11)	0.58 (0.11)	0.10
**eGFR, mL/min per 1.73m**^**2**^	86.9 (16.8)	84.6 (12.7)	86.1 (18.9)	89.2 (17.6)	0.09
**Baseline CRP, mg/L**	4.7 (1.4,16.4)	0.7 (0.4,1.6)	3.7^d^ (1.9,7.9)	16.3^d^^,^^e^ (8.0,28.7)	<0.0001
**3 months CRP, mg/L**	3.8 (1.2,12.3)	0.7 (0.4,1.3)	3.0^d^ (1.5,5.5)	13.1^d^^,^^e^ (6.8,24.2)	<0.0001
**6 months CRP, mg/L**	3.5 (1.1,11.6)	0.7 (0.4,1.3)	2.4^d^ (1.4,4.7)	12.2^d^^,^^e^ (6.5,24.9)	<0.0001
**Comorbid conditions**					
**Hypertension, n (%)**	149 (43)	32 (32)	37 (37)	80 (56) ^d^^,^^e^	0.0003
**Diabetes mellitus, n (%)**	33 (10)	6 (6)	10 (10)	17 (12)	0.30
**Dyslipidemia, n (%)**	100 (29)	28 (27)	25 (25)	47 (33)	0.41
**Overweight, n (%)**	95 (28)	22 (22)	25 (25)	48 (33)	0.11
**Smoking, n (%)**	37 (11)	9 (9)	9 (9)	19 (13)	0.46
**Systolic blood pressure, mmHg**	127 (17)	123 (17)	127 (18)	130 (16) ^d^	0.008
**Diastolic blood pressure, mmHg**	76 (11)	74 (11)	75 (11)	78 (11) ^d^	0.03
**Fasting blood glucose, mg/dl**	94 (18)	91 (12)	94 (21)	96 (19) ^d^	0.06
**Total cholesterol, mg/dL**	195 (32)	198 (30)	194 (34)	195 (31)	0.70
**Low-density lipoprotein cholesterol, mg/dL**	112 (28)	112 (26)	110 (33)	114 (25)	0.43
**High-density lipoprotein cholesterol, mg/dL**	61 (17)	63 (14)	61 (17)	61 (18)	0.59
**Triglyceride, mg/dL**	93 (71,123)	93 (69,123)	93 (72,123)	94 (72,123)	0.79
**Body mass index, kg/ m**^**2**^	23.3 (3.7)	22.8 (3.2)	22.9 (3.3)	23.9 (4.2)	0.04
**Renin-angiotensin system inhibitor, n (%)**	32 (9)	7 (7)	5 (5)	20 (14) ^d^	0.04
**Statin, n (%)**	26 (8)	7 (7)	9 (9)	10 (7)	0.81
**RA-related**					
**Disease duration of RA, years**	6 (3,11)	5 (3,9)	7 (2,11)	6 (3,13) ^d^	0.09
**Rheumatoid factor positive, n (%)**	284 (82)	69 (68)	85 (85) ^d^	130 (90) ^d^	<0.0001
**MTX, n (%)**	187 (54)	40 (40)	58 (58) ^d^	89 (62) ^d^	0.002
**Other DMARDs**	233 (68)	60 (59)	68 (68)	105 (73)	0.08
**Nephrotoxic DMARDs**	177 (51)	49 (49)	54 (54)	74 (51)	0.74
**Biological agent, n (%)**	31 (9)	6 (6)	8 (8)	17 (12)	0.26
**Corticosteroids, n (%)**	192 (56)	33 (33)	61 (61) ^d^	98 (68) ^d^	<0.0001
**NSAIDs, n (%)**	158 (46)	31 (31)	50 (50) ^d^	77 (53) ^d^	0.001

Abbreviations: CRP, C-reactive protein; eGFR, estimated glomerular filtration rate;

RA, rheumatoid arthritis; MTX, methotrexate; DMARDs, disease modifying anti-rheumatic drugs; NSAIDs, nonsteroidal anti-inflammatory drugs.

Data are expressed as mean ±standard deviation, medians (25^th^, 75^th^), or number (%).

Group 1 ^a^ was defined as all three CRP values were ≤3.0 mg/L.

Group 2 ^b^ was defined as one or two CRP values were >3.0 mg/L.

Group 3 ^c^ was defined as all three CRP values were >3.0 mg/L.

^d^ p < 0.05 vs group1.

^e^ p < 0.05 vs group2.

### Outcome according to the presence of baseline CRP subgroups

**Fig 2:**
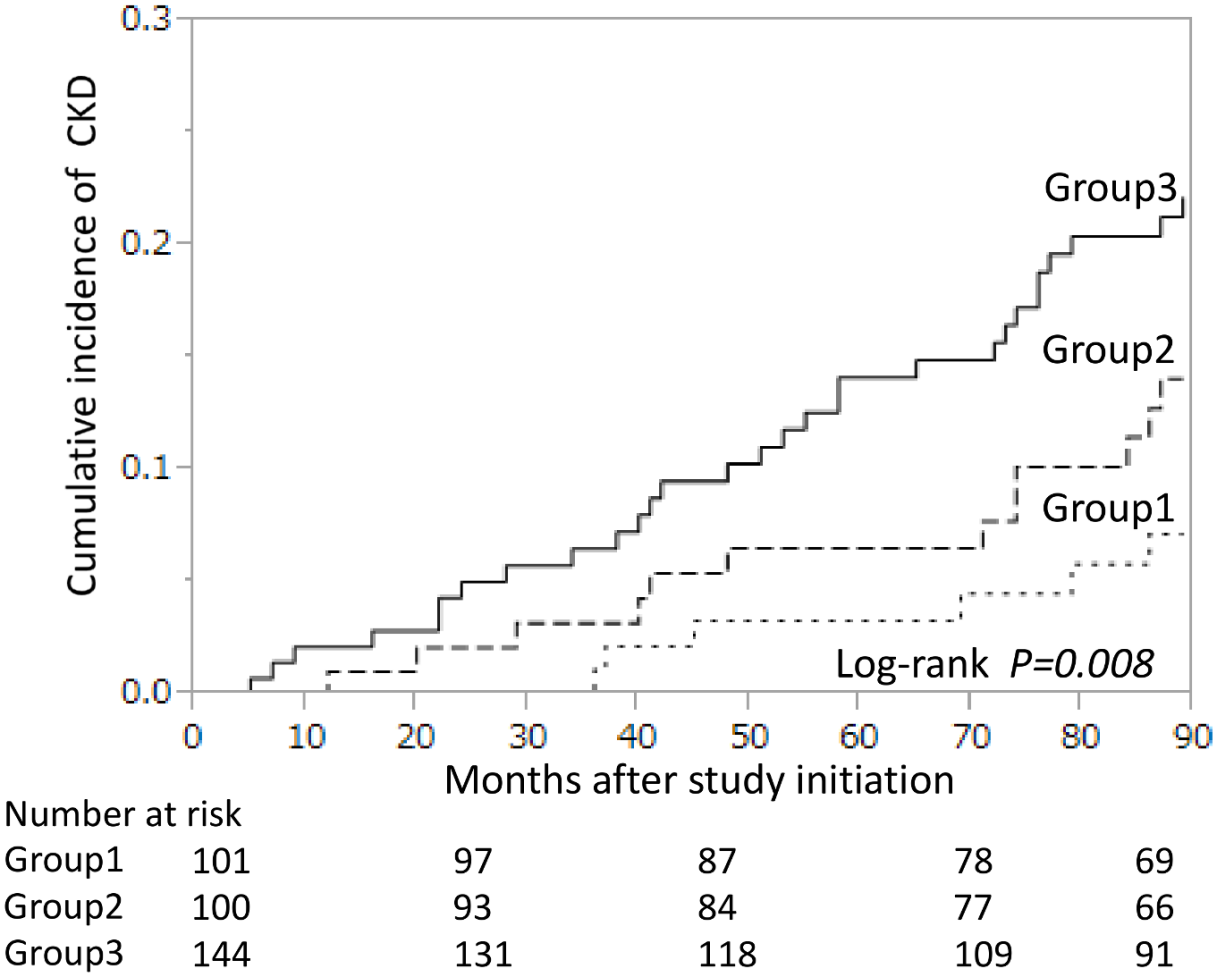
Cumulative incidence of CKD for each C-reactive protein (CRP) subgroup. Observation was started from the third measurement of CRP. Short-dashed line, group 1 (no elevation of CRP values defined as all three CRP values ≤3.0 mg/L); long-dashed line, group 2 (transient high CRP values defined as one or two CRP values >3.0 mg/L); solid line, group 3 (persistently high CRP values defined as all three CRP values >3.0 mg/L). The differences between groups were compared using a log-rank test.

The median follow-up period from the third measurement of CRP was 89 months (range, 1–89 months). Over this period, 16 patients (5%) died, 22 (6%) were lost to follow-up and 41 (12%) were unable to follow-up due to changing hospital. A total of 47 patients (14%) developed CKD (eGFR <60 mL/min/1.73 m^2^ without proteinuria, n = 37; proteinuria without eGFR <60 mL/min/1.73 m^2^, n = 8 and eGFR <60 mL/min/1.73 m^2^ with proteinuria, n = 2). Fig 2 shows the cumulative incidence rates according to baseline CRP subgroups. During the follow-up period, the cumulative incidence of CKD was 7% in group 1, 14% in group 2 and 22% in group 3 by the end of the follow-up period, representing a significant difference among the three groups (P = 0.008, log-rank test).

### Risk of incidence of CKD according to CRP subgroups

As shown in Table 2, incidence of CKD was significantly associated with age, sex, eGFR, CRP subgroups, hypertension, diabetes mellitus, overweight, NSAIDs use at baseline for this study. In contrast, MTX was a negative independent predictor of incidence of CKD.

**Table 2:** Univariate analyses of predictors for incidence of CKD.

Variable	HR (95%CI)	p Value
**Age (every 10 years)**	2.22 (1.70–2.94)	<0.0001
**Male**	2.41 (1.23–4.46)	0.01
**Baseline eGFR (+5ml/min/1.73 m**^**2**^**)**	0.80 (0.71–0.90)	0.0001
**CRP subgroups**		
**Group1**^**a**^	1.00 (reference)	
**Group2**^**b**^	2.04 (0.79–5.85)	0.14
**Group3**^**c**^	3.50 (1.56–9.33)	0.002
**Hypertension**	3.54 (1.91–6.95)	<0.0001
**Diabetes mellitus**	3.43 (1.66–6.52)	0.002
**Dyslipidemia**	1.16 (0.61–2.11)	0.63
**Overweight**	1.94 (1.07–3.46)	0.03
**Smoking**	1.83 (0.79–3.72)	0.14
**Disease duration of RA (1_year increment)**	1.02 (0.99–1.04)	0.17
**Rheumatoid factor positive**	0.99 (0.49–2.29)	0.99
**MTX**	0.44 (0.24–0.79)	0.005
**Nephrotoxic DMARDs**	1.09 (0.62–1.95)	0.76
**NSAIDs**	1.88 (1.06–3.45)	0.03

Abbreviations: CKD, chronic kidney disease; HR, hazard ratio; CI, confidence interval;

CRP, C-reactive protein; RA, rheumatoid arthritis; MTX, methotrexate; DMARDs, disease modifying anti-rheumatic drugs; NSAIDs, nonsteroidal anti-inflammatory drugs.

Group 1^a^ was defined as all three CRP values were ≤3.0 mg/L.

Group 2^b^ was defined as one or two CRP values were >3.0 mg/L.

Group 3^c^ was defined as all three CRP values were >3.0 mg/L.

Table 3 shows HRs for the association between the incidence of CKD and CRP subgroups based on different models using Cox proportional hazard models. In the model 1 adjusted for age and sex, persistently high CRP (group3) was significantly associated with incidence of CKD (HR, 2.72; 95% CI, 1.20–7.28; P = 0.01). The HRs remained significant with the inclusion of baseline eGFR in model 2 and with the inclusion of classical risk factors for CKD in model 3 (hypertension, diabetes mellitus, dyslipidemia, overweight and smoking). In model 4, we also additional adjusted for baseline medication use which may have a toxic effect on the kidney (MTX, nephrotoxic DMARDs and NSAIDs) to model 3 and associations remained significant (HR, 3.00; 95% CI, 1.23–8.53; P = 0.01).

**Table 3:** Multivariate adjusted Hazard ratios for incidence of CKD.

	Model 1	Model 2	Model 3	Model 4
	HR (95% CI)	HR (95% CI)	HR (95% CI)	HR (95% CI)
**CRP subgroups**				
** Group1**^**a**^	1.00 (reference)	1.00 (reference)	1.00 (reference)	1.00 (reference)
** Group2**^**b**^	1.56 (0.60–4.50)	1.54 (0.59–4.47)	1.76 (0.67–5.10)	1.96 (0.70–6.06)
** Group3**^**c**^	2.72 (1.20–7.28)	2.96 (1.31–7.95)	2.63 (1.14–7.16)	3.00 (1.23–8.53)

Abbreviations: CKD, chronic kidney disease; HR, hazard ratio; CI, confidence interval;

CRP, C-reactive protein.

Group 1^a^ was defined as all three CRP values were ≤3.0 mg/L.

Group 2^b^ was defined as one or two CRP values were >3.0 mg/L.

Group 3^c^ was defined as all three CRP values were >3.0 mg/L.

Variables used for adjustment

Model 1; Age (every 10 years) and sex.

Model 2; Model 1 and baseline eGFR (+5ml/min per 1.73 m^2^).

Model 3; Model 2 and comorbidities (hypertension, diabetes, dyslipidemia, overweight and smoking).

Model 4; Model 3 and drugs (MTX, nephrotoxic DMARDs and NSAIDs).

Table 4 shows the results of the multivariate adjusted HRs and p values of all covariates for incidence of CKD in model 4. Incidence of CKD was significantly associated with age, persistently high CRP, hypertension, and use of NSAIDs at study baseline. In contrast, there was a significant inverse association between MTX use and incidence of CKD. Persistently high CRP levels had the strongest impact on the incidence of CKD among the potential risk factors.

**Table 4:** Multivariate analyses of predictors for incidence of CKD.

Variable	HR (95% CI)	p Value
**Age (every 10 years)**	1.92 (1.39–2.65)	<0.0001
**Male**	2.13 (0.91–4.63)	0.07
**Baseline eGFR (+5ml/min/1.73 m**^**2**^**)**	0.94 (0.84–1.04)	0.24
**CRP subgroups**		
**Group1**^**a**^	1.00 (reference)	
**Group2**^**b**^	1.96 (0.70–6.06)	0.20
**Group3**^**c**^	3.00 (1.23–8.53)	0.01
**Hypertension**	2.14 (1.09–4.41)	0.03
**Diabetes mellitus**	1.63 (0.78–3.17)	0.19
**Dyslipidemia**	0.95 (0.48–1.78)	0.87
**Overweight**	1.10 (0.58–2.05)	0.77
**Smoking**	1.14 (0.40–2.93)	0.80
**MTX**	0.34 (0.17–0.66)	0.001
**Nephrotoxic DMARDs**	0.65 (0.34–1.25)	0.20
**NSAIDs**	2.12 (1.12–4.11)	0.02

Abbreviations: CKD, chronic kidney disease; HR, hazard ratio; CI, confidence interval;

CRP, C-reactive protein; MTX, methotrexate; DMARDs, disease modifying anti-rheumatic drugs; NSAIDs, nonsteroidal anti-inflammatory drugs.

Group 1^a^ was defined as all three CRP values were ≤3.0 mg/L.

Group 2^b^ was defined as one or two CRP values were >3.0 mg/L.

Group 3^c^ was defined as all three CRP values were >3.0 mg/L.

### Correlation between the CRP values during the first 6 months of follow-up and those of entire follow-up period

**Fig 3:**
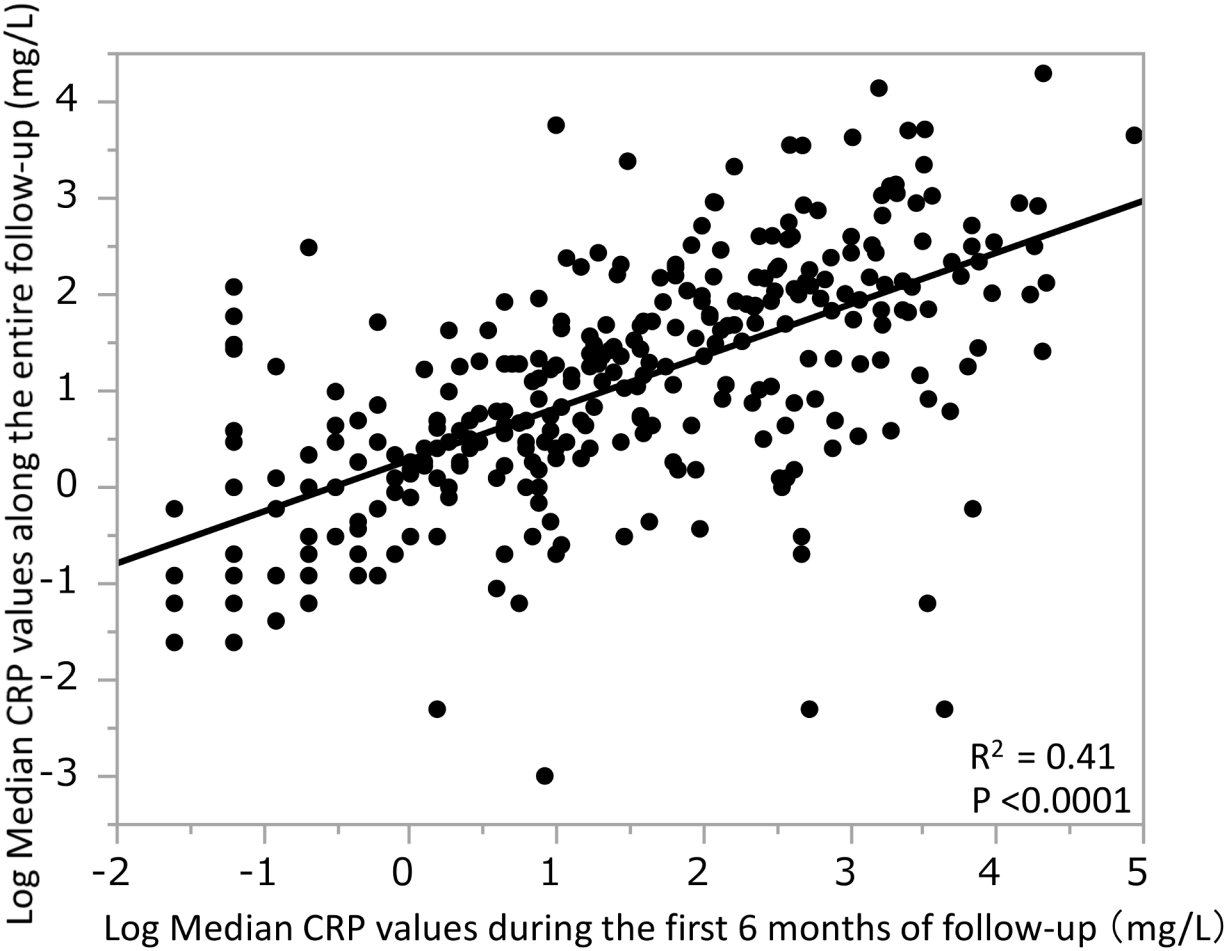
Correlation between the median CRP values along the entire follow-up (log transformed) and the median CRP values during the first 6 months of follow-up (log transformed). The median CRP values along the entire follow-up (log transformed) were positively correlated with the median CRP values during the first 6 months of follow-up (log transformed).

It was not clear whether the CRP levels during the first 6 months of follow-up represented the CRP levels along the entire follow-up. Therefore, we examined the relationship between the median CRP values along the entire follow-up and the CRP values during the first 6 months of follow-up using Pearson’s correlation coefficient with log-transformation of variables because of non-normal distribution of values. (Fig 3). There was a significant positive correlation between CRP levels during the first 6 months and levels across the entire follow-up period (R^2^ = 0.41, p < 0.0001).

### Risk of incidence of CKD according to the median CRP values along the entire follow-up

**Fig 4:**
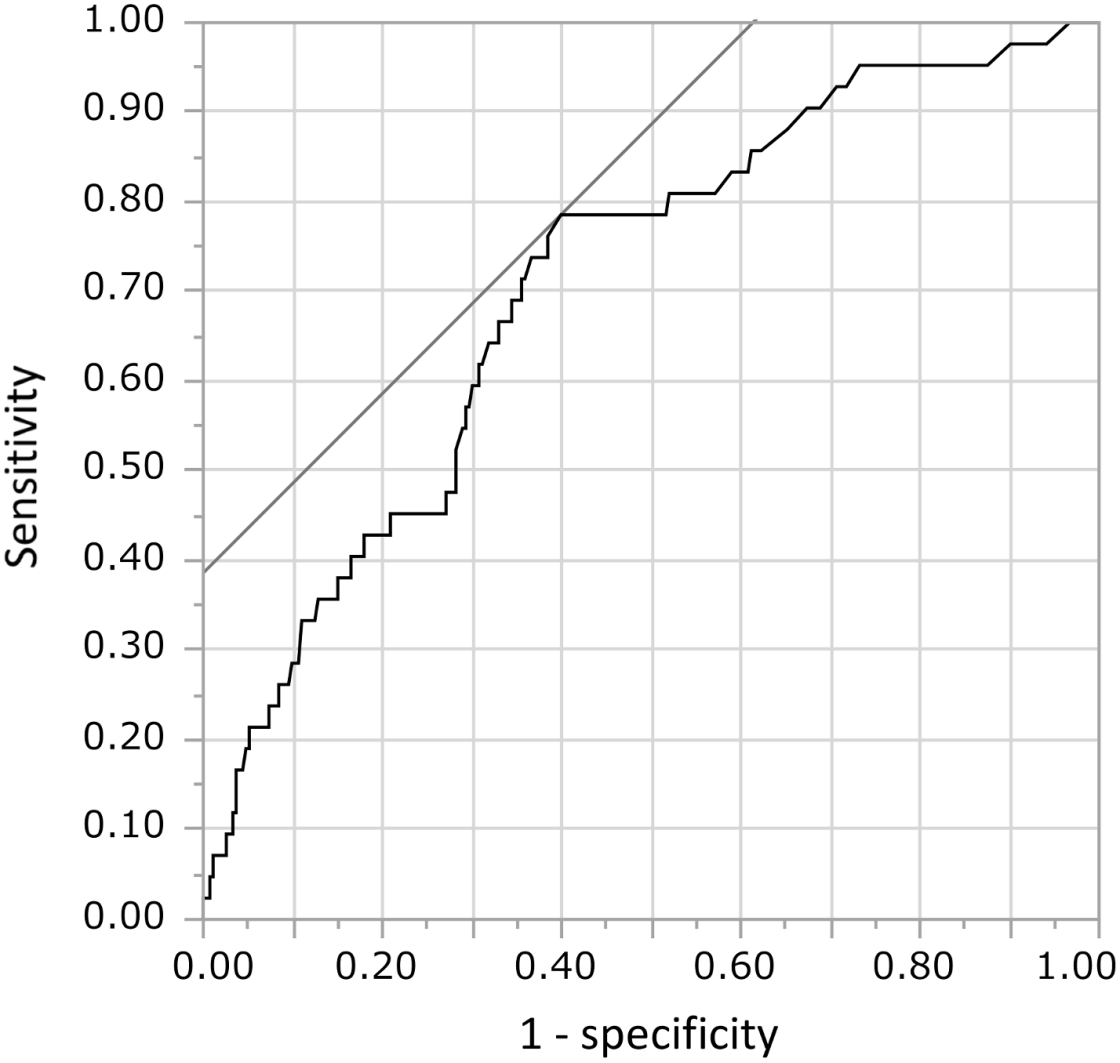
Receiver operating characteristics (ROC) curve for the median CRP values along the entire follow-up in predicting CKD. Sensitivity and specificity were 78.6% and 60.1% at 3.6 mg/L, the most adequate cut-off point of the median CRP values along the entire follow-up. The area under the curve was 0.707.

It is important to define the clinically relevant levels of CRP during the entire follow-up for increasing risk of CKD. We used the area under the curve of the median CRP values along the entire follow-up to predict CKD. Clinically significant inflammation for prediction of incidence of CKD was defined as CRP > 3.6 mg/L, based on the ROC curve (Fig 4).

We further analyzed an association between incidence of CKD and the median CRP values along the entire follow-up using Cox proportional regression analysis. On the basis of the median CRP values along the entire follow-up, patients were divided into two groups: Low CRP (defined as median CRP values along the follow-up of ≤3.6 mg/L) and High CRP (defined as median CRP values along the follow-up of >3.6 mg/L). As shown in Table 5, High CRP was significantly associated with incidence of CKD (HR, 3.69; 95% CI, 1.85–7.99; P = 0.0001) independent of age and sex (model 1). HRs remained significant even after additional adjustment with baseline eGFR (model 2) and with some classical risk factors for CKD (model 3). In model 4, additional adjustment to model 3 with medication use, which may have nephrotoxicity, did not affect its significance (HR, 3.82; 95% CI, 1.86–8.51; P = 0.0002). These results were similar to the data obtained from the baseline CRP subgroups analysis during the first 6 months of follow-up.

**Table 5:** Multivariate adjusted hazard ratios for incidence of CKD according to the median CRP values along the entire follow-up.

	Model 1	Model 2	Model 3	Model 4
	HR (95% CI)	HR (95% CI)	HR (95% CI)	HR (95% CI)
**CRP subgroups**				
** Low CRP** ^**a**^	1.00 (reference)	1.00 (reference)	1.00 (reference)	1.00 (reference)
** High CRP** ^**b**^	3.69 (1.85–7.99)	3.82 (1.92–8.29)	3.88 (1.93–8.50)	3.82 (1.86–8.51)

Abbreviations: CKD, chronic kidney disease; HR, hazard ratio; CI, confidence interval;

CRP, C-reactive protein.

Low CRP ^a^ was defined as median CRP values along the follow-up of ≤3.6 mg/L.

High CRP ^b^ was defined as median CRP values along the follow-up of > 3.6 mg/L.

Variables used for adjustment

Model 1; Age (every 10 years) and sex.

Model 2; Model 1 and baseline eGFR (+5ml/min per 1.73 m^2^).

Model 3; Model 2 and comorbidities (hypertension, diabetes, dyslipidemia, overweight, and smoking).

Model 4; Model 3 and drugs (MTX, nephrotoxic DMARDs and NSAIDs).

## Discussion

Previous studies conducted within the general population have not demonstrated an association between elevated CRP and the incidence of CKD[16, 17]. To the best of our knowledge, the present study is the first to provide evidence showing that persistent elevation of CRP for at least 6 months is a significant risk factor for the development of CKD in patients with RA from a retrospective cohort setting with 89-months follow-up period.

In a population-based study, several markers of inflammation, such as interleukin-6 and tumour necrosis factor-alpha receptor 2 levels, predicted declining kidney function[16–18]. Meanwhile, these studies reported that a single measurement of CRP did not predict a risk of developing an eGFR <60 mL/min/1.73 m^2^ [16, 17]. RA is a chronic inflammatory disease characterised by unpredictable fluctuations in disease activity, and CRP is an acute-phase reactant protein produced in many inflammatory conditions. Thus, measuring its level at a single time point may not be reflective of the true association between the persistently high CRP and incidence of CKD.

Several mechanisms might be involved in the association between inflammation and incidence of CKD. First, a previous study showed that elevated CRP was positively related to blood pressure, glucose, lipids and BMI in a dose-dependent fashion among non-diabetic patients with CKD[19]. Therefore, the relationship between elevated CRP and higher risk for incidence of CKD might be such confounding by risk factors.

Second, renal involvement can be caused by drugs for RA. Patients with RA need often long term therapy with the drugs that are associated with nephrotoxicity such as NSAIDs and DMARDs. Chronic use of NSAIDs is associated with declining eGFR in RA patients[20]. In DMARDs, gold, penicillamine or bucillamine can be associated with proteinuria[21, 22]. Calcineurin inhibitors, such as cyclosporine and tacrolimus, can induce declining GFR[23]. Therefore, toxic effects of these drugs might be responsible for an association between persistent high CRP and incidence of CKD. However, in this study, persistently high levels of CRP were a significant predictor for developing CKD after adjustment for these drugs. Thus, persistent inflammation might have an independent role in the development of CKD.

Third, it is well known that patients with longstanding inflammation may develop secondary amyloidosis in RA[24, 25]. On the other hand, chronic inflammation may directly promote kidney injury by inducing inflammation in the glomerulus and tubulointerstitium. Inflammation reportedly contributes to glomerular injury via infiltration of inflammatory cells such as monocytes and macrophages, which stimulate the proliferation of mesangial cells, leading to renal scarring[26–29]. Furthermore, CRP itself might contribute to the initiation of renal inflammation and fibrosis. A previous report showed deposition of CRP in the glomerular endothelium and cytoplasm of tubules in human kidney biopsies[30, 31]. Human C-reactive protein transgenic mice developed severe renal inflammation with a significant increase in the infiltration of T cells and macrophages into the tubulointerstitium accompanied by the upregulation of proinflammatory cytokines, chemokines and adhesion molecule[32].

Alternatively, alteration in glomerular hemodynamic derived from endothelial dysfunction might be responsible for the CRP-related increased risk for incidence of CKD. It has been reported that CRP was independently associated with endothelial dysfunction in patients with RA[33], and proteinuria was associated with inflammation and endothelial dysfunction[34]. Moreover, inflammation has been suggested to mediate afferent arteriolar structural changes, which could impair the autoregulatory system and result in glomerular hypertension[35]. In accordance with this hypothesis, CRP was significantly associated with hyperfiltration, which is generally reflected by proteinuria among non-diabetic patients with kidney disease[19]. In addition, we previously reported that CRP was associated with prevalent and incident proteinuria in a screened cohort[36]. Thus, elevated CRP in some patients might relate to the incidence of CKD defined as eGFR < 60 mL/min/1.73 m^2^ and/or positive dipstick testing for proteinuria in association with a hyperfiltration state.

Our results have important clinical implications. The prevalence of CKD has been shown to be higher in patients with RA than that in the general population, and its causes are likely to be multifactorial[37, 38]. However, the results of the present study suggest that inflammation might have a pivotal role in the progression of CKD. Thus, tight control of inflammation in RA may provide additional benefits for preventing the development of CKD. This notion was supported by the finding that MTX, which have potential nephrotoxicity, remains as a protective factor rather than a risk factor for incidence of CKD even after adjustment with confounding factors.

In patients with RA, CVD is a leading cause of mortality[39–41]. The European League Against Rheumatism (EULAR) recommends that adequate control of disease activity is necessary to lower the CVD risk[42]. MTX has been associated with a decreased risk of CVD in RA[43, 44]. Moreover, MTX reduced atherogenesis in association with anti-inflammatory effect in an animal atherosclerosis model [45]. Therefore, the anti-inflammatory properties of MTX were suggested to be responsible for reduced risk of CVD. CKD is a potential risk factor for CVD in patients with RA[38, 46], as well as in general population[47–49]. Therefore, protective effects of MTX for incidence of CKD shown in the present study may be benefit for the prevention of CVD in RA. Further studies are warranted to evaluate whether control of inflammation in RA improves CVD-related outcomes through prevention of the development of CKD.

This study had several limitations. First, we used data from dipstick testing for proteinuria but not albuminuria for evaluating urinary protein. Methods for evaluating urinary protein vary between countries; in Japan, dipstick testing for proteinuria is common, and we previously demonstrated its power for predicting cardiorenal outcomes in the general population[50]. Kidney Disease: Improving Global Outcomes (KDIGO) also accepts dipstick testing of proteinuria as a valid method of evaluating urinary protein[51]. Measuring albuminuria is not reimbursed for RA patients. Second, we did not take into account changes in various factors over the course of the study, including medication use which may have a toxic effect on the kidney, RA disease activity, or control status of traditional risk factors, and these undefined factors may affect results. However, the presence of persistently high CRP from the baseline predicted future CKD development regardless of variations in these clinical conditions. It may be unlikely that the same medication use including DMARDs was maintained for during the all follow-up period in all patients. Hence, it may be inadequate to assign CKD to a drug that can be withdrawn several months later. However, drugs including DMARDs and NSAIDs that have been used for a certain period of time can induce drug induced kidney disease. Most forms of parenchymal kidney injury due to drug toxicity can progress to CKD even if the suspected drug-related nephrotoxicity was discontinued[52]. Therefore, medication use which may have a toxic effect on the kidney was used as a confounding factor in this study. Third, it was not clear that the CRP levels during the first 6 months of follow-up represented the levels of CRP during the entire follow-up. We found significant correlation between them, and predictive value for incidence of CKD was comparable. It is clinically useful that evaluation of CRP levels in relatively short time period is predictive for future risk of incident CKD. Fourth, we could not estimate the disease activity, such as Disease activity score (DAS28). CRP is commonly used as a biomarker of systemic inflammation and is included surrogate marker of disease activity. Moreover, CRP is well recognized as predictors of disease outcome[53–55]. Despite the weakness of only CRP in the first 6 months of follow-up as a parameter, we observed a significant correlation between this parameter and incidence of CKD. Fifth, we have not studied about the causes of CKD precisely in this study. The cause of CKD was diabetes in 11%, secondary amyloidosis in 4%, membranous nephropathy in 4% and unknown in majority of the patients. In most patients of unknown cause of CKD, high CRP values were persisted. Therefore, inflammation seems to have significant role in the incident CKD independent of specific renal disease, although we cannot completely exclude the specific renal disease due to the defect of histological analysis by kidney biopsy. Future study is needed that includes a detailed clinical and pathological evaluation. Sixth, this study enrolled a relatively small number of all Japanese patients from a single centre and there are only 47 incident CKD. We cannot exclude that our multivariate model is overfitted, but the resulting estimates do not seem unstable. Therefore, caution must be exercised when extrapolating our results to other patient sets and populations.

In conclusion, the present study shows that persistently high CRP for at least 6 months is a significant risk factor for CKD development, independent of classical risk factors in patients with RA. Results suggest that inflammation is important for the development of CKD and that strict control of inflammation is expected to have clinical benefits including the reduction of incidence of CKD in patients with RA.
